# Validation of discrete time‐to‐event prediction models in the presence of competing risks

**DOI:** 10.1002/bimj.201800293

**Published:** 2019-07-31

**Authors:** Rachel Heyard, Jean‐François Timsit, Leonhard Held, COMBACTE‐MAGNET consortium

**Affiliations:** ^1^ Department of Biostatistics at the Epidemiology Biostatistics and Prevention Institute University of Zurich Hirschengraben Switzerland; ^2^ UMR 1137 IAME University Paris‐Diderot, Inserm Paris France

**Keywords:** area under the curve, calibration slope, competing events, discrete time‐to‐event model, dynamic prediction models, prediction error, validation

## Abstract

Clinical prediction models play a key role in risk stratification, therapy assignment and many other fields of medical decision making. Before they can enter clinical practice, their usefulness has to be demonstrated using systematic validation. Methods to assess their predictive performance have been proposed for continuous, binary, and time‐to‐event outcomes, but the literature on validation methods for discrete time‐to‐event models with competing risks is sparse. The present paper tries to fill this gap and proposes new methodology to quantify discrimination, calibration, and prediction error (PE) for discrete time‐to‐event outcomes in the presence of competing risks. In our case study, the goal was to predict the risk of ventilator‐associated pneumonia (VAP) attributed to *Pseudomonas aeruginosa* in intensive care units (ICUs). Competing events are extubation, death, and VAP due to other bacteria. The aim of this application is to validate complex prediction models developed in previous work on more recently available validation data.

## 1 INTRODUCTION

Clinical prediction models aim to give valid outcome predictions for new patients and to provide a good basis for treatment decisions. Such models need to be systematically validated before entering clinical practice. Assessing the predictive performance in the data set from which the model has been derived will most certainly give an assessment which is too optimistic. To avoid this issue, some kind of cross‐validation or external validation is needed. In perfect conditions, the performance of the prediction model is assessed in a second independent data set (Steyerberg, 2009). Such validation data, also referred to as testing data, should incorporate new patients from a different time period or patients from a different center. To quantify how well the prediction model performs, commonly used measures of discrimination and calibration can be computed. A model has satisfactory discrimination if it is able to adequately discriminate between cases and controls. Moreover, a well‐calibrated model guarantees good agreement between observed outcomes and predictions. Finally, to evaluate overall performance, quadratic scoring rules like the prediction error (PE) or Brier score (BS) can be calculated to simultaneously assess calibration and discrimination.

Heyard, Timsit, Essaied, Held, Heyard et al. (2019) developed dynamic clinical prediction models for discrete time‐to‐event data with competing risks. They extended standard objective Bayesian variable selection methodology to handle discrete‐time competing risks models and identified the most relevant predictors for the timing of ventilator‐associated pneumonia (VAP) caused by a specific organism, *Pseudomonas aeruginosa* (PA). They accounted for the fact that the occurrence of a VAP PA at a particular day can be precluded by the competing events death and extubation as well as by the occurrence of a VAP caused by a different pathogen (noPA). Daily information on intensive care unit (ICU) patients was used from the start of ventilation until the first competing event is observed. With a landmark approach, the authors further developed dynamic prediction models so that different predictors can be used depending on the time already spent at risk. For each landmark, here specified as the day of ventilation, a separate prediction model with distinct regression coefficients is defined. Being able to identify low‐ and high‐risk patients for VAP PA will improve medical practice, targeting the right preventive treatments for these patients. To further enhance the dynamic prediction models, cause‐specific variable selection (CSVS) was performed to account for the fact that some variables might not have a direct effect on a specific outcome but rather an indirect effect by influencing one or more of the competing events.

The use of discrete time‐to‐event methods (Singer & Willett, 1993) was a natural choice for Heyard et al. (2019) due to the daily ICU records in the data. These methods are asymptotically equivalent to the Cox regression (with time‐dependent variables) for short time intervals and small event probabilities in these intervals. A formal proof, as well as several numerical examples can be found in D'Agostino et al. (1990), who refer to discrete time‐to‐event models as pooled logistic regression. Moreover, lagged time‐dependent variables are easily incorporated in discrete‐time models. Allowing for lagged explanatory variables in a clinical prediction model is important as some treatment or medical measurement may not have an immediate effect on the risk of event on the same day, but rather a delayed influence.

To introduce some notation, let $T_{i}$ and $C_{i}$ denote the discrete event and censoring times of subject *i* with support {1,2,⋯}. Furthermore, let $R_{i}\in \left\{1,\cdots ,m\right\}$ denote the cause of event observed for subject *i*. In a time‐to‐event setting with competing risks, the observed event times $t_{i}=\min\left\{T_{i},C_{i}\right\}$ are reported together with censoring indicators

$$\delta_{i}=\left\{\begin{array}{cc} 1 & T_{i}\leq C_{i}\\ 0 & T_{i}>C_{i}\; \end{array}\right.$$

and the observed cause of event $r_{i}\in \left\{1,\cdots ,m\right\}$, with $r_{i}=0$ if and only if $\delta_{i}=0$.

The discrete‐time cause‐specific hazard function (Fahrmeir, 2005) for cause *r* and subject *i* at time *t* is defined as a conditional probability and is modeled using a multinomial logit model:

(1) $$\begin{array}{ccc} \lambda_{r}\left(t\;|\;x_{it}\right) & = & \mathrm{Pr}(T_{i}=t,R_{i}=r\;|\;T_{i}\geq t,x_{it})\\ & = & \frac{\exp(\beta_{0tr}+x_{it}^{⊤}\beta_{r})}{1+\sum_{j=1}^{m}\exp\left(\beta_{0tj}+x_{it}^{⊤}\beta_{j}\right)},\;\text{for}\;r=1,\cdots ,m,\;t=1,2,\cdots , \end{array}$$

where $\beta_{0tr}$ is the cause‐specific time‐dependent intercept, $\beta_{r}$ is the cause‐specific vector of coefficients, and $x_{it}$ is the covariate vector. Note that a reference category needs to be defined (cause r=0). In most applications, this refers to “staying at risk”. To use multinomial logit models in practice, the data have to be reformatted in a way that for a subject with observed event time $t_{i}$ we define $y_{is}=\left(y_{is0},y_{is1},\cdots ,y_{ism}\right)=\left(1,0,\cdots ,0\right)$ for all $s=1,\ldots ,t_{i}-1$. If the event $r_{i}$ is observed for observation *i* at time $t_{i}$ (so $\delta_{i}=1$), we define

$$y_{it_{i}}=\left(y_{it_{i}0},y_{it_{i}1},\cdots ,y_{it_{i}m}\right)=\left(0,\cdots ,1,\cdots ,0\right),$$

with $y_{it_{i}r_{i}}=1$ and all other elements of $y_{it_{i}}$ set to zero. If subject *i* is censored at time $t_{i}$ (so $r_{i}=0$ and $\delta_{i}=0$), we define

$$y_{it_{i}}=\left(y_{it_{i}0},y_{it_{i}1},\cdots y_{it_{i}m}\right)=\left(1,0,\cdots ,0\right).$$

Berger and Schmid (2018) refer to this data structure as the *augmented set of observations*.

In the cause‐specific hazard (1), $\beta_{0tr}$, t=1,2,⋯, represent the cause‐specific baseline coefficients and are estimated, together with $\beta_{r}$, using maximum likelihood (ML). Objective Bayesian methods based on the generalized *g*‐prior are used to shrink the ML estimates toward zero (Held, Bové, & Gravestock, 2015; Held, Gravestock, & Bové, 2016).

In this paper, we will first motivate our research by introducing the application on the OUTCOMEREA database in Section 2. Then, in Section 3, we will present the methods used for the validation of dynamic discrete‐time competing risks prediction models. Afterward, in Section 4, these methods are applied to the data presented in Section 2. Finally, Section 5 closes with some discussion.

## 2 CASE STUDY

VAP is the leading nosocomial infection in critically ill ventilated patients treated in an ICU and PA is the most common organism associated with VAPs (Hunter, 2012). Heyard et al. (2019) were interested in the time until a first occurrence of a VAP attributed to PA. However, once ventilator‐assisted patients are extubated or dead they are not at risk for a VAP PA anymore. Furthermore, the occurrence of a VAP PA and a VAP attributed to a different bacterium (VAP noPA) are mutually exclusive events. We are thus in the presence of competing risks. Heyard et al. (2019) developed dynamic competing risks models to answer their research question. They further developed a method for CSVS to simplify their models and account for the fact that some variables may not have a direct effect on each outcome. Using Bayesian methodology they decided to set some of the cause‐specific coefficients to zero and corrected the remaining coefficients accordingly. The data used for the development of these dynamic cause‐specific prediction models are an extract from the OUTCOMEREA database (Bekaert et al., 2011; Bouadma et al., 2015; Truche et al., 2016) with information on patients admitted to 32 French ICUs between January 1997 and August 2015. To apply discrete time‐to‐event methods, the data have to be coded in an augmented format as discussed in the previous section. The patients are analyzed from start of ventilation until the occurrence of a first event and represented in the data set by as many rows as they stay ventilated. This training data set on which the models were developed contains information on 5,123 distinct ICU stays constituting 64,164 ventilation‐days.

Before the models can be used in clinical practice they need to be validated. To do so, we will use more recent information from the OUTCOMEREA database with patients admitted to the ICU between beginning of 2015 and end of 2017. This external testing data set contains 5,733 ventilation‐days for 534 distinct ICU admissions. Table 1 gives the number of distinct events in both data sets. Since a patient needs to be at least extubated before leaving the ICU, no censoring is present in these data. Note that there is an overlap between the periods for the training and testing data. This is due to the fact that the information of some of the patients admitted in early 2015 was not fully recorded in the database when the training set was extracted.

**Table 1:** Total number of distinct events in the training and testing data

	Dead	Extubated	VAP noPA	VAP PA
Training data	896	3,251	635	341
Testing data	126	312	72	24

Notes:. The information of the patients is only analyzed until the occurrence of a first event.

The probability of interest is $\mathrm{Pr}(T_{i}=t+2,R_{i}=r\;|\;T_{i}\geq t,x_{it})$, the risk of an event *r* happening two days after the prediction time point *t*, given that patient *i* is still at risk at day *t* and the possibly time‐dependent covariate vector $x_{it}$. Predicting two days ahead allows the physician to better target treatment with, for example, specific antibiotics. As discussed in Heyard et al. (2019), this probability is obtained by

$$\mathrm{Pr}\left(T=t+2,R=r\;|\;T\geq t,x_{t}\right)=\lambda_{r}\left(t+2\;|\;x_{t}\right)\cdot \left[1-\lambda \left(t\;|\;x_{t}\right)\right]\cdot \left[1-\lambda \left(t+1\;|\;x_{t}\right)\right],$$

with $\lambda_{r}\left(\cdot \right)$ being the cause‐specific hazard and λ(·) the overall hazard.

## 3 VALIDATION OF DYNAMIC DISCRETE‐TIME COMPETING RISKS PREDICTION MODELS

Evaluating the predictive performance of a model in the same data set in which it has been developed most likely gives an assessment that is too optimistic. To achieve external validation, the model is developed in a training data set and assessed in a testing data set independent of the first one. See Steyerberg (2009) for more information on internal and external validation.

Henderson, Jones, and Stare (2001) indicate that in time‐to‐event analysis, point predictions such as the expected residual lifetime (in the case of fatal absorbing endpoints) are subject to serious error. Hence, the focus here is on probabilistic predictions, for example, the predicted risk of an event at a certain time point. Standard validation methods for binary prediction models can be extended to discrete time‐to‐event models. Assessing the performance of a prediction model means that the ability to discriminate between cases and controls (discrimination) but also the statistical agreement between observations and predictions (calibration) needs to be considered.

In the time‐to‐event setting, cases and controls are defined at specific points in time. A subject can then be a case at time *t* while being a control at time $t^{\prime }<t$. A censored subject stays a control over the whole observation period. The PE quantifies the distance between predicted and observed incidences and examines calibration and discrimination simultaneously. A good prediction model should perform well in all those aspects. The researcher should not only focus on a single measure since good discrimination does not guarantee good calibration and vice versa. The next sections will explain how discrete‐time competing risks models are evaluated with respect to their calibration, discrimination, and overall performance.

### 3.1 Discrimination

In a clinical setting, discrimination refers to a model's ability to distinguish between patients with and without a specific endpoint. Discrimination measures consider time‐to‐event outcomes as time‐dependent binary outcomes with levels “event at *t*” and “event after *t*”. Hence, existing methods for binary classification rules can be used. Extensions of such established concepts to assess the discriminative performance include the time‐dependent area under the receiver operating characteristics curve (AUC). Methods to estimate this dynamic AUC over time have been discussed for single outcome continuous time‐to‐event data (see Heagerty & Zheng, 2005) and discrete‐time models (Schmid, Tutz, & Welchowski, 2018). The continuous‐time methods have recently been extended to models allowing for time‐dependent covariates and competing risks, see, for example, Saha and Heagerty (2010), Zheng, Cai, Jin, and Feng (2012), Blanche, Dartigues, and Jacqmin‐Gadda (2013), and Blanche et al. (2015). Li, Greene, and Hu (2018) and van Houwelingen and Putter (2012) present simpler closed‐form methods to estimate the time‐dependent AUC without the need for numerical integration. We will extend these techniques which have been developed for single outcome models with baseline variables only.

Let $\pi_{ir}\left(t\right)$ be the risk prediction for subject *i* at time point *t*, with $\pi_{ir}\left(t\right)=0$ for all subjects who are not at risk at time *t*. The predictions are cause‐specific, meaning that separate time‐dependent AUCs will be computed for each event *r* and $\pi_{ir}\left(t\right)$ refers to the risk prediction of cause *r* at *t*, as for example the cause‐specific hazard $\lambda_{r}\left(t\;|\;x_{it}\right)$. Furthermore, let

(2) $$D_{ir}\left(t\right)=1\left\{T_{i}=t,R_{i}=r\right\}$$

indicate whether an event of cause *r* was observed at time *t* for subject *i* or not. The cause‐specific dynamic AUC,

(3) $${\text{AUC}}_{r}\left(t\right)=\mathrm{Pr}\left(\pi_{ir}\left(t\right)>\pi_{jr}\left(t\right)\;|\;D_{ir}\left(t\right)=1,D_{jr}\left(t\right)=0,T_{i}\geq t,T_{j}\geq t\right)$$

represents the probability of a random case *i* with observed event *r* at time *t* having a higher risk prediction for cause *r* than a randomly selected control *j* without the observed event *r* at time *t* (adapted from Blanche et al., 2015). If the true event status at time point *t* for each subject at risk is observed, we can follow Li et al. (2018) to estimate (3) by generalizing their approach to discrete‐time competing risks models with time‐dependent variables and predictions:

(4) $${\hat{\text{AUC}}}_{r}\left(t\right)=\frac{\sum_{i=1}^{n}\sum_{j=1}^{n}\left[1\left\{\pi_{ir}\left(t\right)>\pi_{jr}\left(t\right)\right\}+0.5\cdot 1\left\{\pi_{i}\left(t\right)=\pi_{j}\left(t\right)\right\}\right]D_{ir}\left(t\right)\left(1-D_{jr}\left(t\right)\right)}{\sum_{i=1}^{n}\sum_{j=1}^{n}D_{ir}\left(t\right)\left(1-D_{jr}\left(t\right)\right)}.$$

The term $0.5\cdot 1\{\pi_{i}\left(t\right)=\pi_{j}\left(t\right)\}$ accounts for occasional ties in risk predictions, which are commonly observed in discrete‐time data. In the case of censoring, the event indicators in (2) may not be known for each *t* and *i*. In this case, Li et al. (2018) suggest to use weights for each subject estimated with a kernel‐weighted Kaplan–Meier method and replace (2) in (4) by their weight estimates. Alternatively, Blanche et al. (2015) and Schmid et al. (2018) use inverse probability of censoring weight (IPCW) estimates.

As a time‐independent summary measure of discriminative power, the discrete‐time cause‐specific version of the *C*‐index can be estimated as a weighted sum of (4) (Tutz & Schmid, 2016):

(5) $${\hat{C}}_{r}=\sum_{t\in T}{\hat{\text{AUC}}}_{r}\left(t\right)\cdot {\hat{w}}_{r}\left(t\right)$$

with

(6) $${\hat{w}}_{r}\left(t\right)=\hat{\mathrm{Pr}}\left(T=t,R=r\right)\cdot \hat{\mathrm{Pr}}\left(T>t\right)/\sum_{u\in T}\hat{\mathrm{Pr}}\left(T=u,R=r\right)\cdot \hat{\mathrm{Pr}}\left(T>u\right).$$

The set T contains the time points for which the AUC is evaluated and integrated over. Schmid et al. (2018) used similar weights (without competing risks) which were adopted here. They proved that, in the case of a single outcome model and a baseline marker or linear predictor $\eta_{i},i=1,\cdots ,n$ their definition of the *C*‐index equals the probability $\mathrm{Pr}(\eta_{i}>\eta_{j}\;|\;T_{i}<T_{j})$ which is the global concordance index, known as Harrell's C (Harrell, Lee, & Mark, 1996). The estimates $\hat{\mathrm{Pr}}\left(T=t,R=r\right)$ and $\hat{\mathrm{Pr}}\left(T>t\right)$ are computed using the Kaplan–Meier estimates of the cause‐specific hazards ${\hat{\lambda }}_{r}\left(t\right)$:

$$\begin{array}{ccc} \hat{\mathrm{Pr}}\left(T=t,R=r\right) & = & {\hat{\lambda }}_{r}\left(t\right)\prod_{s=1}^{t-1}\left(1-\sum_{r=1}^{m}{\hat{\lambda }}_{r}\left(s\right)\right)\\ \hat{\mathrm{Pr}}\left(T>t\right) & = & \prod_{s=1}^{t}\left(1-\sum_{r=1}^{m}{\hat{\lambda }}_{r}\left(s\right)\right). \end{array}$$

As a time‐ and cause‐independent measure, a summary of discriminative performance can be computed using a weighted sum of the estimated cause‐specific *C*‐indices:

(7) $$\hat{C}=\sum_{r=1}^{m}{\hat{C}}_{r}\cdot e_{r}/e,$$

where $e_{r}$ is the number of events of cause *r* and $e=\sum_{r}e_{r}$, for each time point t∈T. This quantity gives a model an overall score, which makes model comparisons easier. Finally, the closer (4), (5), and (7) are to 1, the better the predictions are discriminated. To account for the uncertainty of the AUC estimate, Li et al. (2018) suggest to use bias‐corrected accelerated bootstrap confidence intervals (Efron & Tibshirani, 1994). This method is rather time‐consuming and induces sampling error. We will compute so‐called jackknife standard errors (see Section 3.4) to quantify the uncertainty of the quantities introduced here, as also done in Antolini, Boracchi, and Biganzoli (2005). Note that the way the cases for the time‐dependent AUC were defined is similar to the definition of incident cases in Saha and Heagerty (2010) as opposed to the alternative of cumulative cases. For the incident cases, a subject is a case at *t* if he or she had an observed event at *t*. Cumulative cases have an event *by* time point *t*.

If the focus is on dynamic predictions, the ${\text{AUC}}_{r}\left(t\right)$ can be conditioned on being at risk at an earlier time point s≤t (Blanche et al., 2015):

(8) $${\text{AUC}}_{r}^{\mathrm{cond}}\left(s,t\right)=\mathrm{Pr}\left(\pi_{ir}\left(s,t\right)>\pi_{jr}\left(s,t\right)\;|\;D_{ir}\left(s,t\right)=1,D_{jr}\left(s,t\right)=0,T_{i}>s,T_{j}>s\right).$$

In the discrete time‐to‐event scenario, without censoring, the dynamic and conditional AUC_*r*_ is estimated using $\pi_{ir}\left(s,t\right)=\mathrm{Pr}\left(T_{i}=t,R_{i}=r\;|\;T_{i}>s,x_{is}\right)$ and $D_{ir}\left(s,t\right)=1\left\{T_{i}=t,R_{i}=r\;|\;T_{i}>s\right\}$ in (4). To compute the conditional *C*‐index, conditional event probabilities have to be used for the weights.

### 3.2 Calibration

Besides discrimination, another important aspect on which prediction models are evaluated is calibration, which informs on the reliability of the predicted risks. To assess the calibration performance of our models, we will use the calibration plot (CP) as well as the calibration slope (CS) and intercept (CI) (Steyerberg, 2009). Again, cause‐specific versions of these quantities are computed. The cause‐specific CP compares the cause‐specific hazards ${\hat{\lambda }}_{r}\left(t\;|\;x_{it}\right),i=1,\cdots ,n,t=1,\cdots ,T_{i}$ of the model to the relative frequencies of observed events of cause *r* in predefined subsets of the augmented data. Berger and Schmid (2018) propose an approach for a single outcome time‐to‐event model. Their plot is very similar to the one for a binary outcome model. For a fixed cause *r* and each subject and time point, the hazards are fitted. Then the latter are split into, for example, 10 or 20 subsets $G_{kr}$, defined by the percentiles of the estimated hazards. The relative frequencies of the observed events are computed in each subset by $|G_{kr}{|}^{-1}\sum_{i,t}D_{ir}\;1\left\{{\hat{\lambda }}_{r}\left(t\;|\;x_{it}\right)\in G_{kr}\right\}$, where $|G_{kr}|$ is the number of observations in $G_{kr}$. In a next step, these fitted and observed event frequencies are plotted against each other to assemble a CP. In a well‐calibrated model, the pairs are close to the diagonal line. van Hoorde et al. (2014) present an alternative method to visualize the calibration of multinomial logistic regression, which we will not further discuss here (an example can be found in the supplementary material).

For the CS and CI, we will follow the approach by van Hoorde et al. (2014), whose parametric solution to estimate the observed proportions is to use the results from the logistic recalibration framework:

(9) $$\log\left(\frac{\lambda_{r}\left(t\;|\;x_{it}\right)}{\lambda_{0}\left(t\;|\;x_{it}\right)}\right)=a_{r}+\sum_{j=1}^{m}b_{r,j}\eta_{ij},$$

where the multinomial time‐dependent outcome is regressed on the linear predictors $\eta_{ij}=\beta_{0tj}+x_{it}^{⊤}\beta_{j}$ derived from (1). As discussed by these authors, there is no one‐to‐one relationship between the predicted and observed proportions: for a specific value of the prediction ${\hat{\lambda }}_{r}\left(t\;|\;x_{it}\right)$, the predicted hazards of the other m−1 causes (not *r*) might vary, which leads to different values for $\eta_{ir}$ and therefore for the observed proportions by the means of (9).

A model is perfectly calibrated if the intercepts $a_{r}$ for r=1,⋯,m in (9) are all zero, the slopes $b_{r,r}$ are one and the slopes $b_{r,j;j\neq r}$ are zero. We will retain the $b_{r,r},r=1,\cdots ,m$ as the cause‐specific calibration slopes (CS_*r*_), quantifying the risk of overfitting (if ${\text{CS}}_{r}<1$). The $a_{r}$ are retained as the cause‐specific calibration intercepts (CI_*r*_), representing calibration‐in‐the‐large. The latter is an overall population‐level measure that informs on the difference between observed and average risk predictions. For estimation of the key parameters $b_{r,r}$ for the CS_*r*_ and $a_{r}$ for the CI_*r*_, the linear predictors $\eta_{ij,j\neq r}$ can be considered as nuisance parameters as they do not involve cause *r*. To obtain an estimate of CS_*r*_, the noncorresponding slopes $b_{r,j\neq r}$ in Equation (9) are set to 0 (using constraints in the R function vglm{VGAM}). To estimate CI_*r*_, $a_{r}$ can be computed by fixing $b_{r,r}$ and $b_{r,j\neq r}$ to 1 and 0, respectively. It is straightforward to compute 95% Wald confidence intervals for the CS and CI using the respective standard errors of the recalibration framework in (9) with specific constraints. As an overall check for calibration, we propose a likelihood ratio test with 2·m degrees of freedom to test the joint null hypothesis that $a_{r}=0,b_{r,r}=1\;|\;b_{r,j\neq r}=0$ in (9). If this null hypothesis is rejected, separate tests (with *m* degrees of freedom) for the intercepts and slopes can be considered. To implement all these methods, the supplementary material of van Hoorde et al. (2014) was a good resource. Note that these calibration measures do not have to be corrected for censoring because, in the case of censoring, the observed relative frequencies and the estimated hazards decrease by a similar amount.

### 3.3 Prediction error

To examine calibration and discrimination simultaneously, Graf, Schmoor, Sauerbrei, and Schumacher (1999), Schumacher, Graf, and Gerds (2003), and van Houwelingen and Putter (2012) suggest a time‐dependent version of the BS to assess the predictive performance. The BS has originally been developed to judge the inaccuracy of probabilistic weather forecasts (Brier, 1950). Gerds and Schumacher (2006) show how this score can be consistently estimated using IPCW in the presence of censored data. Later, Schoop, Graf, and Schumacher (2008) and Schoop, Schumacher, and Graf (2011b) extend the loss function from the previous papers to adequately deal with time‐dependent variables and dynamic predictions. They refer to their score as the PE. The PE is the expected time‐dependent quadratic loss, the difference between the observed event status and the predicted probabilities by the model at a specific time point *t*.

In a competing risks setting, we are forced to compute cause‐specific PEs and follow Schoop, Beyersmann, Schumacher, and Binder (2011a) by adapting their approach to the discrete‐time framework. The predicted probability of an event of cause *r* happening by time *t*, also known as the cumulative incidence function of cause *r*, is defined as ${\text{CIF}}_{r}\left(t\right)=\mathrm{Pr}\left(T\leq t,R=r\right)$ (Beyersmann, Allignol, & Schumacher, 2012). The discrete‐time cause‐specific PE at time *t* is then

(10) $${\text{PE}}_{r}\left(t\right)=\frac{1}{n}\sum_{i=1}^{n}{\left[\;1\left\{T_{i}\leq t,R_{i}=r\right\}-{\text{CIF}}_{ri}\left(t\right)\;\right]}^{2},$$

with $1\{T_{i}\leq t,R_{i}=r\}$ being an indicator for subject *i* of experiencing an event of cause *r* by time *t*. Given that we estimate the discrete‐time cause‐specific hazard function ${\hat{\lambda }}_{r}\left(t\;|\;x_{it}\right),r=1,\cdots ,m$ of subject *i* as described in the introduction, the CIF of cause *r* for subject *i* with (time‐dependent) covariate vector $x_{it}$ is estimated as follows:

(11) $${\hat{\text{CIF}}}_{ri}\left(t\;|\;x_{it}\right)=\sum_{u=1}^{t}\hat{\mathrm{Pr}}\left(T_{i}>u-1\;|\;x_{iu}\right)\;{\hat{\lambda }}_{r}\left(u\;|\;x_{iu}\right),$$

$$\text{with}\;\hat{\mathrm{Pr}}\left(T_{i}>u-1\;|\;x_{iu}\right)=\prod_{v=1}^{u-1}\left(1-\sum_{k=1}^{m}{\hat{\lambda }}_{k}\left(v\;|\;x_{iv}\right)\right).$$

To account for censored observations, IPCW is again used for estimation, as already discussed for the AUC. The PE is represented as a curve depending on time. A “naive” prediction model, with an event probability of .5 for each *t*, would give a PE equal to .25, so we aim for the estimated cause‐specific PE curve to be smaller than .25 at each time‐point. However, if events are very rare, the predictions of a “naive” model such as the null model will be very small too, leading to a PE much smaller than .25. Note that, to compute the CIF${}_{r}\left(t\right)$, the hazards for cause *r* up to time *t* have to be computed for all subjects even if they have experienced a competing event k≠r. This does not pose a problem if only baseline covariates are used for the predictions. However, with time‐dependent variables it is more complicated, since their values will be unknown after the subject experienced a competing event. We will come back to this issue in the discussion. For now we simply use the last‐observation‐carried‐forward approach to substitute the missing values.

Following Tutz and Schmid (2016) and generalizing their method to the competing risks setting, the integrated PE curve, a time‐independent coefficient of PE, can be estimated as follows:

(12) $${\hat{\text{IPE}}}_{r}=\sum_{t\in T}{\hat{\text{PE}}}_{r}\left(t\right)\;\hat{\mathrm{Pr}}\left(T=t,R=r\right),$$

where T are the time points for which the PE is evaluated and summed over. The marginal probability $\hat{\mathrm{Pr}}\left(T=t,R=r\right)$ can be computed in the same way as the weights in (5), using Kaplan–Meier estimates of the hazards. The PE curve, as well as the IPE, are negatively oriented meaning that the lower they are, the better the performance of the model. Confidence intervals for the IPE have not been systematically discussed in the literature. One option is to use bootstrap methods for standard errors as well as confidence intervals, but this is quite computer‐intensive. We decided, as already for the *C*‐index to use jackknife standard error estimates to compute CIs for the IPE.

van Houwelingen and Putter (2012) define the relative error reduction (RER), which can directly be used in the discrete‐time competing risks setting

(13) $${\hat{\text{RER}}}_{r}\left(t\right)=\frac{{\text{PE}}_{r}^{\mathrm{null}\;\mathrm{model}}\left(t\right)-{\text{PE}}_{r}^{\mathrm{model}}\left(t\right)}{{\text{PE}}_{r}^{\mathrm{null}\;\mathrm{model}}\left(t\right)},$$

where ${\text{PE}}_{r}^{\mathrm{null}\;\mathrm{model}}\left(t\right)$ is the cause‐specific PE of the null model without any covariates but only the time‐dependent and cause‐specific intercepts at time *t*. ${\text{PE}}_{r}^{\mathrm{model}}\left(t\right)$ is the same score but of the model of interest.

To address dynamic predictions, Schoop et al. (2011b) condition on the fact that the subjects are still at risk at an earlier point in time s≤t. In the discrete time‐to‐event setting, this conditional PE score, denoted by ${\text{PE}}_{r}^{\text{cond}}$, is then estimated by

(14) $${\hat{\text{PE}}}_{r}^{\mathrm{cond}}\left(t,s\right)=\frac{1}{n_{s}}\sum_{i=1}^{n_{s}}{\left[1\left\{T_{i}\leq t,R_{i}=r\;|\;T_{i}>s\right\}-{\hat{\text{CIF}}}_{ri}^{\mathrm{cond}}\left(t,s\right)\right]}^{2},$$

where $n_{s}$ is the number of subjects still at risk at time point *s*. The conditional cause‐specific CIF is estimated by adding the condition $T_{i}>u$ into the two terms in (11) resulting in the following equation:

$${\hat{\text{CIF}}}_{ri}^{\mathrm{cond}}\left(t,s\right)=\sum_{u=s}^{t}\hat{\mathrm{Pr}}\left(T_{i}>u-1\;|\;T_{i}>s,x_{is}\right)\hat{\mathrm{Pr}}\left(T_{i}=u,R_{i}=r\;|\;T_{i}>s,x_{is}\right)/\hat{\mathrm{Pr}}\left(T_{i}>u\right).$$

Furthermore, we have $\hat{\mathrm{Pr}}\left(T_{i}>u-1\;|\;T_{i}>s,x_{is}\right)=\prod_{v=s}^{u-1}[1-\sum_{k=1}^{m}\hat{\mathrm{Pr}}\left(T_{i}=v,R_{i}=k\;|\;T_{i}>s,x_{iv}\right)]$. In the presence of censoring, conditional IPCW weights can be used. Also, a conditional IPE can be computed by summing up the ${\hat{\text{PE}}}_{r}^{\mathrm{cond}}$ as in (12) but using conditional weights, $\hat{\mathrm{Pr}}\left(T=t,R=r\;|\;T>s\right)$.

### 3.4 Jackknife estimate of the standard error

The *jackknife* (Efron, 1982) is a resampling technique commonly used to estimate standard errors of certain parameters. Let $x_{1},\cdots ,x_{n}$ denote the data set used to estimate our parameter θ. Furthermore, let us define the estimate $\hat{\theta }$ based on the whole data set. Let ${\hat{\theta }}_{-i}$ be the estimate of the same parameter based on the data without subject *i* with $\bar{\theta }=\sum_{i=1}^{n}{\hat{\theta }}_{-i}$. Then, the jackknife estimate of the standard error of $\hat{\theta }$ is

$$\sqrt{\frac{n-1}{n}\sum_{i=1}^{n}{\left({\hat{\theta }}_{-i}-\bar{\theta }\right)}^{2}}.$$

This enables us to easily compute standard errors of the *C*‐index and the IPE. The jackknife method is computationally less intensive then the bootstrap which would need a larger number of repetitions; the jackknife only needs *n*. In addition, the approach discussed here does not create any sampling error.

## 4 CASE STUDY REVISITED

Heyard et al. (2019) were interested in predicting the probability of a VAP PA event happening at day t+2 given that the patient is still at risk at day *t*. The cause‐specific hazards at t+2 as well as the overall hazards at t+1 and *t* are used to predict

(15) $$\begin{array}{ccc} \mathrm{Pr}(T=t+2,R=r\;|\;T\geq t,x_{t}) & = & \lambda_{r}\left(t+2\;|\;x_{t}\right)\cdot \left[1-\lambda \left(t\;|\;x_{t}\right)\right]\cdot \left[1-\lambda \left(t+1\;|\;x_{t}\right)\right]\\ & = & \lambda_{r}\left(t+2\;|\;x_{t}\right)\cdot M\left(t\;|\;x_{t}\right). \end{array}$$

Furthermore, a dynamic prediction model for $\lambda_{r}\left(t+2\;|\;x_{t}\right)$ has been proposed using a landmarking approach which we now want to validate. To ensure the robustness of their models, Heyard et al. (2019) computed landmark‐specific prediction models only until day or landmark 14 since start of ventilation. To further ensure to still observe some events, we will only predict the cause‐specific hazards until 3 weeks after the start of ventilation. The supplementary material deals with the validation of the conditional probability in (15).

In the following, we compare the prediction performance of the following models for $\lambda_{r}\left(t+2\;|\;x_{t}\right)$ over the three first weeks since start of ventilation. We start with the simplest model and add complexity as we go on.

Model **A** is a static prediction model; at each landmark the same predictors with the same shrunken coefficients are used. This model simply includes the variables selected by Heyard et al. at the first landmark, which is day 5.
Model **B** is a dynamic prediction model; at each landmark a different set of predictors with their respective shrunken coefficients are used.
Model **C** is the final dynamic prediction model selected by Heyard et al. (2019). This is model **B**, but with CSVS.

A more detailed description of the models can be found in the supplementary material, together with Figures representing the model coefficients.

We investigate whether Heyard et al.'s approach of selecting the variables dynamically and even accounting for the fact that some variables may not directly affect specific events (see CSVS in Section 2), improved the predictions. To do this, the ability to discriminate between different events over time, the cause‐specific calibration performance as well as the overall prediction performance of the three candidate models above are evaluated. To ensure that we observe some events at all time points considered, we evaluate the predictions only up to 21 days since start of ventilation. In a first step, the models are developed and fitted on the training data set used in the original paper. The dynamic models (**B** and **C**) are then updated during the two first weeks and the model of landmark 14 is used for the last week of evaluation. In the next step, the cause‐specific hazards are predicted in the validation data, introduced in Section 2, using the coefficients obtained in the first step. Table 2 informs on the number of different causes of event during this 3‐week evaluation period. Comparing Tables 2 and 1 shows that there are more events after day 21 in the testing data, but only four more VAPs.

**Table 2:** The number of distinct daily events in the testing data set during the first 3 weeks of ventilation

	Days since start of ventilation (*t*)																	
Event cause	5	6	7	8	9	10	11	12	13	14	15	16	17	18	19	20	21	$\sum_{t}$
Dead	22	20	5	13	9	7	12	5	3	5	2	0	4	2	2	3	1	115
Extubated	38	52	44	35	25	18	13	15	13	6	3	5	8	4	2	5	1	287
VAP noPA	17	7	11	5	7	4	3	3	3	2	3	3	0	0	0	1	1	70
VAP PA	3	3	6	0	1	1	3	0	1	0	3	0	0	0	0	0	1	22

### 4.1 Discrimination

**Figure 1:**
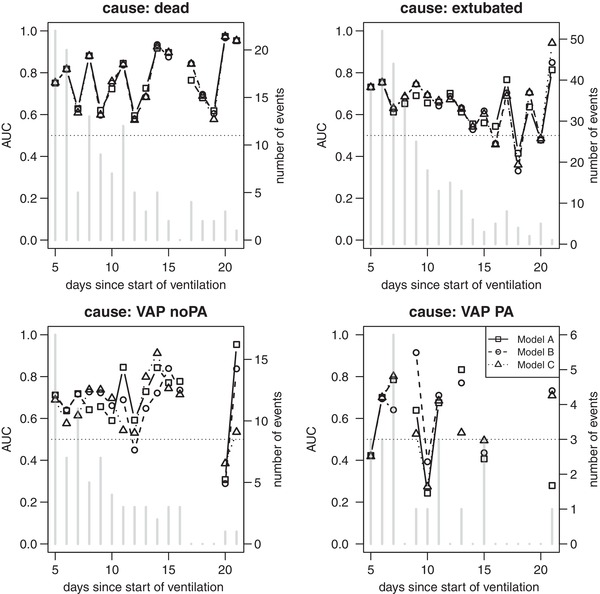
Cause‐specific AUC${}_{r}\left(t\right)$ curves for the three models of interest following Li et al. (2018)

Figure 1 shows the cause‐specific time‐dependent AUC for the three first weeks since start of ventilation, estimated as described in (4). When interpreting this figure, the number of actual events at each time point has to be considered (see bars in the plots). No AUC can be computed for cause *r* if no events of cause *r* are observed at a specific time, which is quite often the case for cause VAP PA. The curves are also not very smooth when only few events are observed. For event causes “dead” and “extubated”, the three models are very similar. The dynamic approach and CSVS do not improve the discriminative power considerably here. For the causes “VAP noPA” and “VAP PA”, the ranking of the models according to their AUC depends strongly on time *t*. This is why the *C*‐index, a time‐independent summary of discriminative performance, might be more informative. To compute the *C*‐index, the missing AUCs at the points in time without an event of a certain cause are set to 0.5, which is the AUC resulting from flipping a coin to decide on the outcome. The weights in the calculation of the *C*‐index at these same time points are very close to 0. The set T in (5) is then defined as T∈{5,6,⋯,21}. Table 3 lists the cause‐specific *C*‐indices for the different models together with their 95% jackknife confidence intervals. All three models perform similarly with model B being slightly better for the VAP causes. The poor performance of the models for “VAP PA” is due to the low daily counts of VAPs in the validation set. Furthermore, since the AUC measure and the *C*‐index do not penalize model complexity, it is not surprising that the static model performs as good as the dynamic models; the static model considers more variables and is therefore more complex for later time points *t*, compared to models **B** and **C**. A null model with only the time‐ and cause‐dependent intercept gives an AUC(*t*) equal to 0.5 for each *t* and cause‐specific, overall *C*‐indices also equal to 0.5. This is due to the definition in Equation (3) and the way ties are accounted for. Table 4 presents a cause‐ and time‐independent measure of discrimination, where the cause‐specific *C*‐indices are summed up using weights depending on the number of final events, as described in (7). According to these numbers, Model **B** would rank first in discrimination, even though the differences are only very small and the uncertainty is quite large.

**Table 3:** The cause‐specific *C*‐indices with their 95% jackknife confidence intervals, for the three prediction models

	Model **A**		Model **B**		Model **C**	
Dead	0.751	[0.70, 0.80]	0.749	[0.70, 0.80]	0.748	[0.69, 0.80]
Extubated	0.689	[0.65, 0.72]	0.700	[0.67, 0.73]	0.701	[0.67, 0.73]
VAP noPA	0.684	[0.63, 0.74]	0.689	[0.63, 0.75]	0.658	[0.60, 0.72]
VAP PA	0.583	[0.41, 0.76]	0.585	[0.41, 0.76]	0.575	[0.41, 0.74]

**Table 4:** The overall cause‐independent *C*‐index (7) with 95% jackknife confidence interval for all models

Model **A**		Model **B**		Model **C**	
0.698	[0.67, 0.72]	0.705	[0.68, 0.73]	0.700	[0.67, 0.73]

### 4.2 Calibration

**Figure 2:**
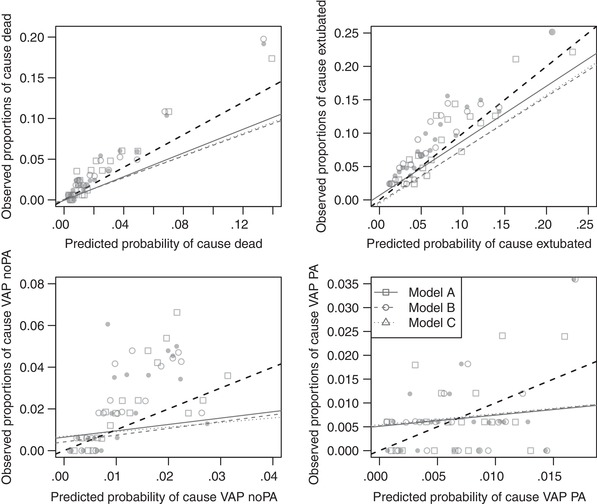
Cause‐specific calibration plots for the different models following Berger and Schmid. The dashed line is the ideal 45 degree line indicating, while the solid lines are simple regression lines

The cause‐specific CPs following the approach by Berger and Schmid (2018) can be found in Figure 2. An alternative way of presenting the CP is shown in the supplementary material. Again, the performance of the models is very different depending on the cause we are interested in. Due to the low event rate of the VAP causes, their predicted cause‐specific probabilities are small too. The pairs of predicted probability and observed proportion should be as close as possible to the ideal diagonal line. All four models are better calibrated for causes “dead” and “extubated” than the VAP outcomes, however they also perform very similar. The cause‐specific CS and CI are more informative and make a comparison between models easier. The CS and CI for each cause and model can be found in Table 5, together with their 95% Wald confidence intervals as well as the *p*‐values of the joint (test 1) and separate (test 2) hypothesis tests of miscalibration. The closer the CS is to 1, the better the calibration of the model, so the better the predictions and the observations agree. Models **C** and **B** are closest for causes “dead” and “VAP PA”. Model **C** performs slightly better for cause “VAP PA”, but very poorly for “VAP PA”. Model **A** is best regarding the cause “extubated”. Apart from the one for cause “VAP no PA” and model **A**, they are all at least slightly smaller than 1, suggesting little‐to‐medium overfitting of the models. The CI should be as close as possible to zero. Here, the three models indicate underestimation of the risks of all causes versus the reference, staying at risk, since all CIs are positive. The overall calibration test rejects the null hypothesis for each model. However, if separate tests are computed on the slopes and the intercepts it turns out that there seems to be only a problem for calibration‐in‐the‐large since separate tests for the intercepts show evidence for miscalibration, while the ones for the slopes do not.

**Table 5:** Cause‐specific calibration slopes and intercepts for the three prediction models with their 95% Wald confidence intervals and the *p*‐values of the joint (test 1) and separate (test 2) likelihood ratio tests as discussed in Section 3.2

	Model **A**		Model **B**		Model **C**	
	CS	CI	CS	CI	CS	CI
Dead	0.97 [0.79; 1.16]	0.39 [0.20; 0.58]	0.98 [0.79; 1.16]	0.45 [0.26; 0.64]	0.98 [0.80; 1.16]	0.45 [0.25; 0.64]
Extubated	0.90 [0.71; 1.09]	0.15 [0.03; 0.27]	0.83 [0.65; 1.01]	0.35 [0.22; 0.47]	0.82 [0.65; 1.00]	0.35 [0.23; 0.47]
VAP noPA	1.12 [0.69; 1.55]	0.55 [0.31; 0.79]	0.94 [0.55; 1.33]	0.73 [0.49; 0.96]	0.94 [0.54; 1.33]	0.69 [0.45; 0.93]
VAP PA	0.69 [0.02; 1.36]	0.07 [−0.35; 0.49]	0.55 [−0.05; 1.14]	0.07 [−0.35; 0.49]	0.69 [0.03; 1.34]	0.04 [−0.38; 0.46]
*p*‐Val (test 1)	<.0001		<.0001		< 0.0001	
*p*‐Val (test 2)	.69	<.0001	.23	<.0001	.32	<.0001

### 4.3 Prediction error

**Figure 3:**
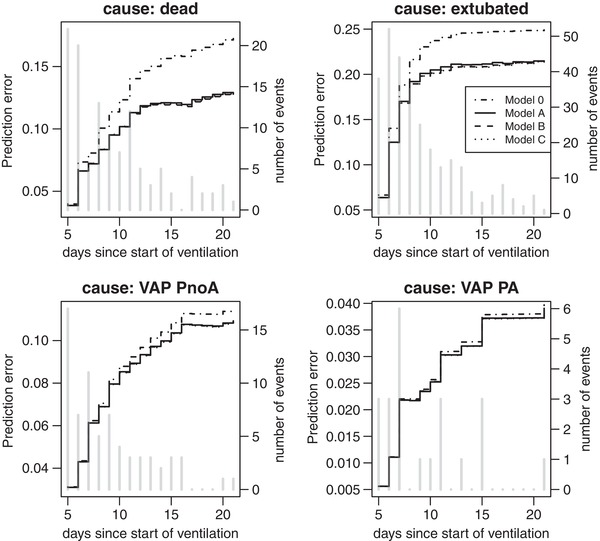
Cause‐specific prediction error curves for the three models of interest, with the number of daily events

**Figure 4:**
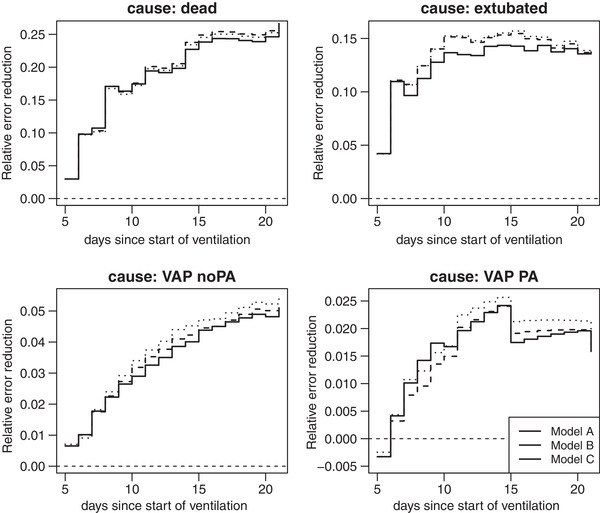
Cause‐specific relative error reduction curves for different prediction models

The PE curves for the 3 weeks since start of ventilation are represented in Figure 3 for each of the competing events. The smaller the PE the better the prediction performance of the model. The three models **A**, **B**, and **C** perform better than chance for all causes as they stay below the PE of the null model for all time points. To get a better overview of the evolution of the scores and on how the three models of interest compare to each other, we compute the PE reduction relative to the null model as in (13). Figure 4 represents these cause‐specific RERs over time. The RER shows to which extend the considered models improve predictions compared to the null model. Now, we can clearly see that model **C** performs best for most time points; only for cause “dead”, model **B** has slightly better performance. In a last step, we compute a time‐independent measure of predictive performance, the cause‐specific integrated PE. These can be found in Table 6 (multiplied by 100) for the three models of interest as well as for the null model without any covariate. We also added 95% confidence intervals based on jackknife estimators of the standard error. The model with the smallest IPE has best performance. This is model **B** for “dead” and model **C** for all the other outcomes “VAP noPA”. For the other outcomes, the models **A**, **B**, and **C** have similar performance but are always better than the null model. However, the CIs are too large to conclude on a significant difference between models, which is due to the small number of daily events in the validation set.

**Table 6:** The cause‐specific integrated prediction errors (× 100) together with their 95% jackknife confidence intervals for the different prediction models

	Model **0**		Model **A**		Model **B**		Model **C**	
Dead	2.188	[1.51; 2.86]	1.815	[1.25; 2.38]	1.807	[1.25; 2.37]	1.813	[1.25; 2.38]
Extubated	10.134	[9.02; 11.25]	8.932	[7.88; 9.98]	8.837	[7.75; 9.92]	8.832	[7.75; 9.92]
VAP noPA	0.877	[0.50; 1.25]	0.853	[0.49; 1.21]	0.852	[0.49; 1.21]	0.850	[0.49; 1.21]
VAP PA	0.089	[0.019; 0.159]	0.088	[0.018; 0.157]	0.088	[0.019; 0.157]	0.088	[0.019; 0.156]

In conclusion, the complex prediction model developed in previous work to predict the diagnosis of a VAP PA at day t+2 using baseline and daily information until day *t* does not perform considerably better than a simplified model in our validation data set. Depending on the cause of interest, either model **B** or model **C** perform best. Regarding calibration, model **A**, the static model, is best for some causes. Further the differences between scores may not be relevant given the low counts, especially for the VAP causes.

## 5 DISCUSSION

In this paper, we suggested methodology to conduct systematical (external) validation of discrete‐time competing risks models. We extended methods that have been proposed either in the continuous time‐to‐event framework or in the context of multinomial logistic regression. We presented a discrete‐time version of the cause‐specific and time‐dependent area under the curve which evaluates the ability of the model to discriminate between cases and controls at different points in time. Moreover, we developed discrete‐time versions of cause‐specific CPs and extracted CSs and CIs to decide whether the predictions and observations agree. Finally, to assess calibration together with discrimination, we demonstrated how discrete‐time PE curves can be computed. These methods were applied to validate complex prediction models previously developed to predict the daily risk of VAP PA diagnosis in ventilated ICU patients. The Bayesian methodology used to develop these prediction models induces optimal shrinkage of the regression coefficients. This improves the predictions, especially if the number of events per variable is small as De Jong et al. (2019) showed in the multinomial logit setting. We were very lucky to acquire up‐to‐date and independent data to make an external validation possible. Unfortunately, the daily counts of events in the testing data are quite small for some time points, so that we are confronted to high uncertainty in the assessment of the performance of our candidate models.

To our knowledge, this is the first paper focusing on the validation of discrete‐time competing risks models. Berger and Schmid (2018) recently illustrated how the calibration performance of single outcome discrete‐time models can be represented. Schmid et al. (2018) worked on discrimination measures for discrete time‐to‐event predictions and provide software to apply them when only baseline variables are included and one cause of event is considered. De Jong et al. (2019) nicely summarize how to assess the prediction performance of multinomial logistic models which we applied to the discrete‐time competing risk framework. Furthermore, a great deal of work has been put into the development of validation methodology in the continuous‐time setting, for single and competing events. However, methods to assess calibration in such models are not discussed systematically. The present paper tries to summarize all the knowledge on validation measures for time‐to‐event predictions, combines them with the literature for multinomial logistic regression, and translates everything to the discrete‐time context. Some tasks remain for future work, as, for example, the lack of easily computable standard errors for the time‐dependent cause‐specific AUCs and PEs.

Another challenge is the computation of the cumulative incidence function. Estimating the CIF is needed to calculate the PE. However, for the CIF of cause *r*, the cause‐specific hazards of the subjects which have observed a competing event (not *r*) need to be computed until the time point *t* for which the PE is evaluated. The values of the included variables have to be known for subjects which are not observed anymore. This is not an issue if only baseline variables are considered but becomes quite complex once time‐varying variables are included. As explained in (Kalbfleisch & Prentice, 2002, pp. 196–200), if these time‐dependent variables are external, they are not affected by the outcome and their path can be defined up to any time *t*. In the case of internal covariates which are related to the continuous‐time hazards, a common assumption is that the covariate process is predictable (Chang & Hsiung, 2005). Kalbfleisch and Prentice (2002) then suggest to model the time‐dependent covariate process jointly with the hazards. The discrete‐time conditional PE (14) is less affected by this problem since only subjects still at risk at time point *t* are included in its computation, see supplementary material for details.

In the case study, the conclusion was that the most complicated model might not be the one with the best prediction performance for some causes. Choosing the best degree of complexity for the final prediction model strongly depends on the cause one is most interested in. Here, the main cause of interest was “VAP PA”, for which only a small number of events were observed in the testing data set. The performance of all models considering this specific cause was not optimal so that future work needs to focus on the optimization of the models with respect to cause “VAP PA”. Maybe it would be worth to consider including new (time‐dependent) variables which have not been included before, but have a considerable effect on “VAP PA”. Furthermore, increasing the sample size of the testing data will also give clearer results.

## Supporting information

Supporting Information

Supporting Information

Supporting Information
