# Supplementary material for: Validation of discrete time‐to‐event prediction models in the presence of competing risks
Source: Biom J. 2019 Jul 31;62(3):643–57. doi: 10.1002/bimj.201800293 (PMC7217187; doi:10.1002/bimj.201800293)
Supplement: Supplementary file 1 — Supporting Information [file BIMJ-62-643-s003.pdf]

Biometrical Journal () , zzz–zzz / DOI: 10.1002/

Supplementary material for *Validation of discrete time-to-event prediction models in the presence of competing risks*

R. Heyard et al

1 Description of models A, B and C

Heyard et al. [2019] applied Bayesian variable selection methodology to the OUTCOMEREA data to predict the timing of a VAP PA, while taking the competing events (death, extubation and VAP noPA) into account. The variables to be selected from are listed in Table 1. They did this in a dynamic way using landmarking, so that depending on the time since start of ventilation different variables may be selected. The Bayesian variable selection methods induce optimal shrinkage of the regression coefficients [Held et al., 2015]. After these dynamic prediction models were fitted, the authors went one step further and applied cause-specific variable selection (CSVS) by setting cause-specific coefficients of variables which did not have a direct effect on the specific cause to zero. The remaining coefficients were then corrected. This very complex model **C** is compared to simpler versions; also see Figure 1 for the shrunken cause-specific regression coefficients of the dynamic prediction model. The coefficients here depend on the timing of the actual prediction. The shrunken time- and cause-specific coefficients *without* CSVS of model **B** are to be found in Figure 2. The coefficients of model **A** can also be found in the last Figure, however, by taking only the one for the first landmark (at day 5), as this model is stationary and the coefficients stay the same, regardless of the timing of the prediction.

2 Calibration plot following van Hoorde et al. [2014]

The calibration plot following the approach presented by van Hoorde et al. [2014] can be found in Figure 3. For the CP of cause r, the predicted cause-specific hazards $\hat{\lambda}_r(t \mid \mathbf{x}_{it})$ are plotted versus the observed proportions or probabilities of cause r. To estimate the observed proportions, the logistic regression framework is used as discussed in Section 3.2 in the manuscript.

3 Assessment of models for the cause-specific two-days-ahead prediction

The aim of the case study in Heyard et al. [2019] was the prediction of the conditional probability $\Pr(T = t+2, R = r \mid T \geq t, \mathbf{x}_t)$. In Section 4 in the paper, the prediction performance of the model for the cause-specific hazard was evaluated. In the following we show how conditional versions of the cause-specific time-dependent AUCs and PEs can be used to assess the performance of the model to predict the probability of interest estimated in equation (15) in the manuscript over time. We use the same prediction models **A**, **B** and **C** for the cause-specific hazards as before. The overall hazards to calculate $M(t \mid \mathbf{x}_t)$ are predicted with very similar models, but adapted to single endpoint time-to-event data. The unique event is then simply 'event of any kind'. Then we compute the conditional quantities $\widehat{\text{AUC}}_r^{\text{cond}}(t+2, t)$ and $\widehat{\text{PE}}_r^{\text{cond}}(t+2, t)$, so that, in our case $s = t - 2$ depends on t.

Figure 4 shows the conditional cause-specific time-dependent AUCs. These plots look very similar to the unconditional ones in the main manuscript and it is hard to decide on a 'winning' model as the ranking by AUC varies much with time and cause. This is why we compute a time-independent discrimination measure, the conditional C-index in Table 2. For causes 'dead' and 'VAP noPA' model **A** has highest C-index while model **B** has best discrimination for causes 'VAP PA' and model **C** for 'extubated'. However, the associated uncertainty is quite large. We finally compute the conditional cause-specific relative error

© WILEY-VCH Verlag GmbH & Co. KGaA, Weinheim

Baseline variables	
x_1	admission type (1=surgical, 0=medical)
x_2	gender (1=male, 0=female)
x_3	Simplified Acute Physiology Score II at first day of admission (0-123)
x_4	admitted with a pneumonia (yes or no)
x_5	sepsis at the admission to the ICU (yes or no)
x_6	ICU admission motif (main symptom): factor with 5 levels
	1: Multiorgan failure - different shocks
	2: Acute respiratory distress syndrome - COPD exacerbation
	3: Acute renal failure
	4: Coma
	5: Continuous monitoring - Scheduled surgery - Trauma (= Reference category)
x_7	diabetes (yes or no)
x_8	at least one comorbidity (yes or no)
**Time-dependent variables with possible lags ($t, t-1, t-2$)**	
$x_{9(t)}$, $x_{9(t-1)}$, $x_{9(t-2)}$	usage of hemodialysis
$x_{10(t)}$, $x_{10(t-1)}$, $x_{10(t-2)}$	presence of a catheter
$x_{11(t)}$, $x_{11(t-1)}$, $x_{11(t-2)}$	daily sequential organ failure assessment score (0-24)
$x_{12(t)}$, $x_{12(t-1)}$, $x_{12(t-2)}$	use of at least one antibiotic (AB) against PA (yes or no)
	Aminoglycosides / Penems / Fosfomycin / Ceftazidime
	Fluoroquinolones / Ureido-carboxypenicillins / Cefpirome/cefepime
$x_{13(t)}$, $x_{13(t-1)}$, $x_{13(t-2)}$	do not resuscitate (yes or no) (DNR)
$x_{14(t)}$, $x_{14(t-1)}$, $x_{14(t-2)}$	colonization or infection with P. aeruginosa resistant
	to at least 2 molecules out of the 3: ticarcillin, ceftazidime or imipenem

Table 1 Potential predictors and their definition.

reduction curves in Figure 5. Our candidate models perform worse than the null model for cause 'dead' whereas the dynamic predictions perform better for all other causes, especially for later t.

	Model **A**		Model **B**		Model **C**	
dead	0.75	[0.67, 0.82]	0.74	[0.67, 0.82]	0.74	[0.67, 0.82]
extubated	0.64	[0.57, 0.70]	0.64	[0.57, 0.71]	0.65	[0.57, 0.72]
VAP noPA	0.67	[0.59, 0.74]	0.65	[0.58, 0.72]	0.64	[0.58, 0.70]
VAP PA	0.52	[0.45, 0.60]	0.53	[0.47, 0.59]	0.52	[0.47, 0.58]

Table 2 The conditional cause-specific C-indices for the three prediction models with their 95% confidence intervals.

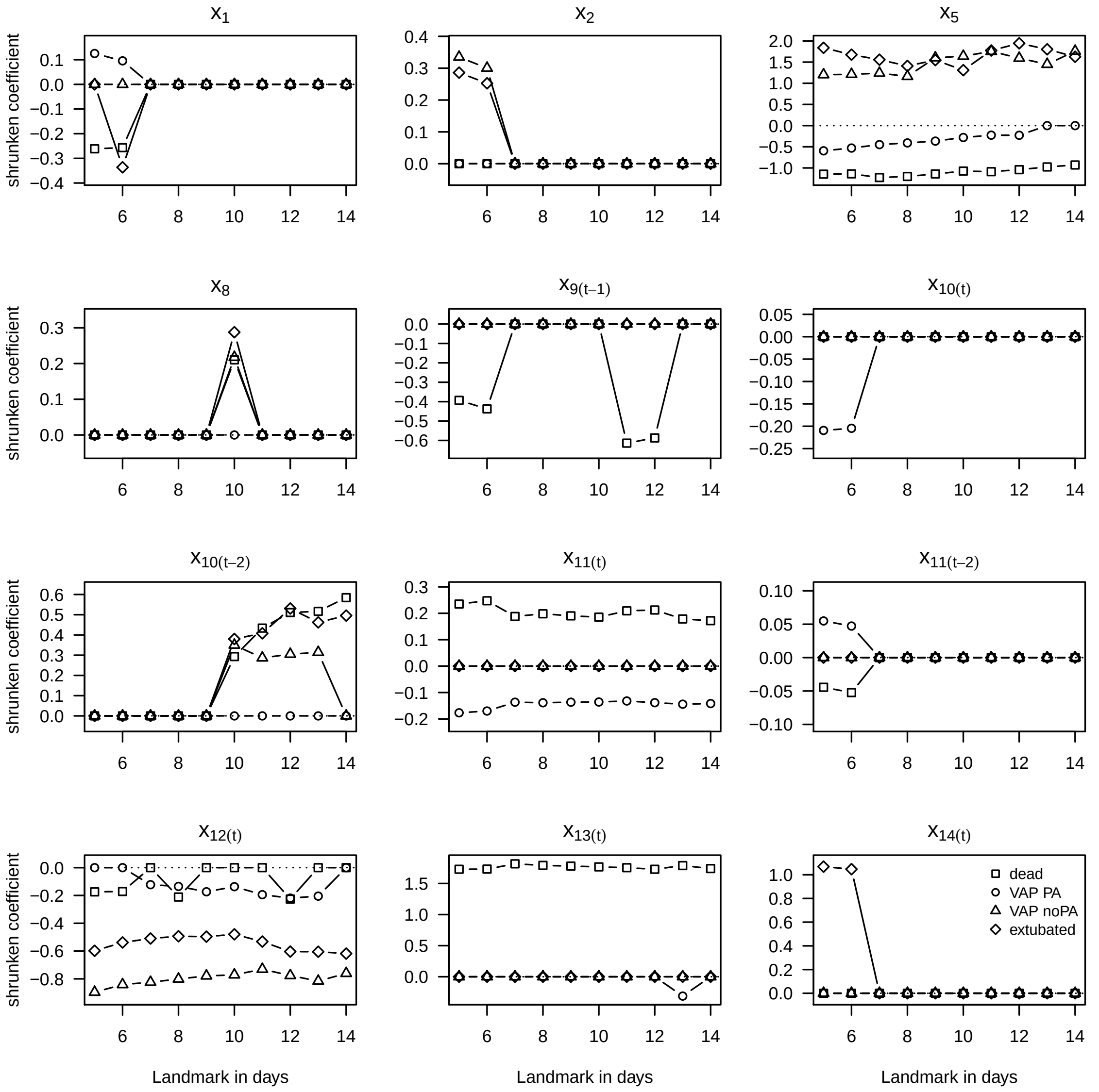

Figure 1 Shrunken cause-specific regression coefficients of the dynamic prediction model **C** after dynamic variable selection via landmarking and cause-specific variable selection. The variables never selected in the variable selection procedure are not present in this figure.

References

L. Held, D. Sabanés Bové, and I. Gravestock. Approximate Bayesian model selection with the deviance statistic. *Statistical Science*, 30(2):242–257, 2015.

R. Heyard, J.-F. Timsit, W. I. Essaied, L. Held, and on behalf of the COMBACTE-MAGNET consortium. Dynamic clinical prediction models for discrete time-to-event data with competing risks—a case study on the outcomerea database. *Biometrical Journal*, 61(3):514–534, 2019. doi: 10.1002/bimj.201700259.

K. van Hoorde, Y. Vergouwe, D. Timmerman, S. van Huffel, E. W. Steyerberg, and B. van Calster. Assessing calibration of multinomial risk prediction models. *Statistics in Medicine*, 33:2585–2596, 2014.

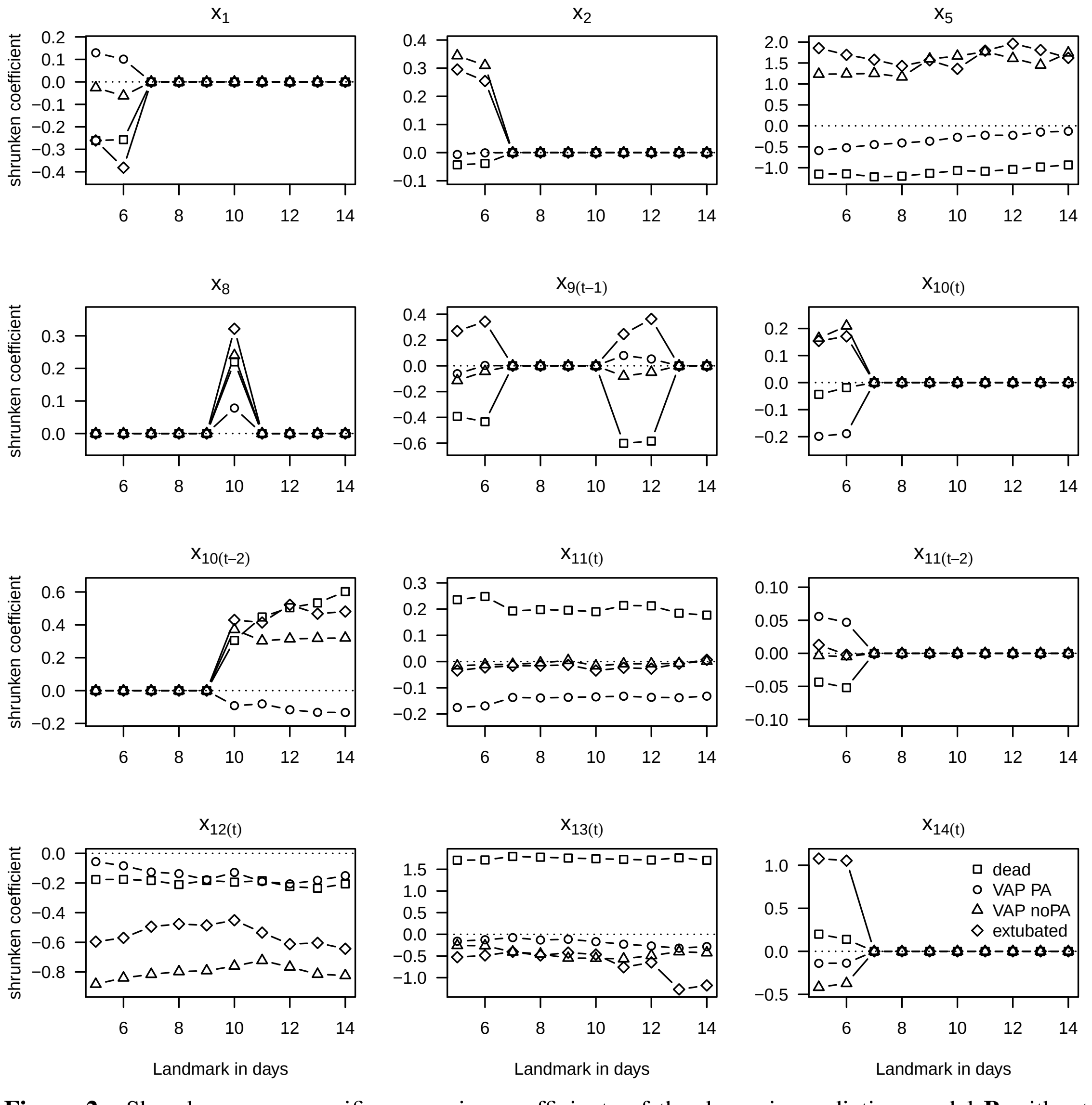

Figure 2 Shrunken cause-specific regression coefficients of the dynamic prediction model **B** without cause-specific variable selection. The variables never selected in the variable selection procedure are not present in this figure.

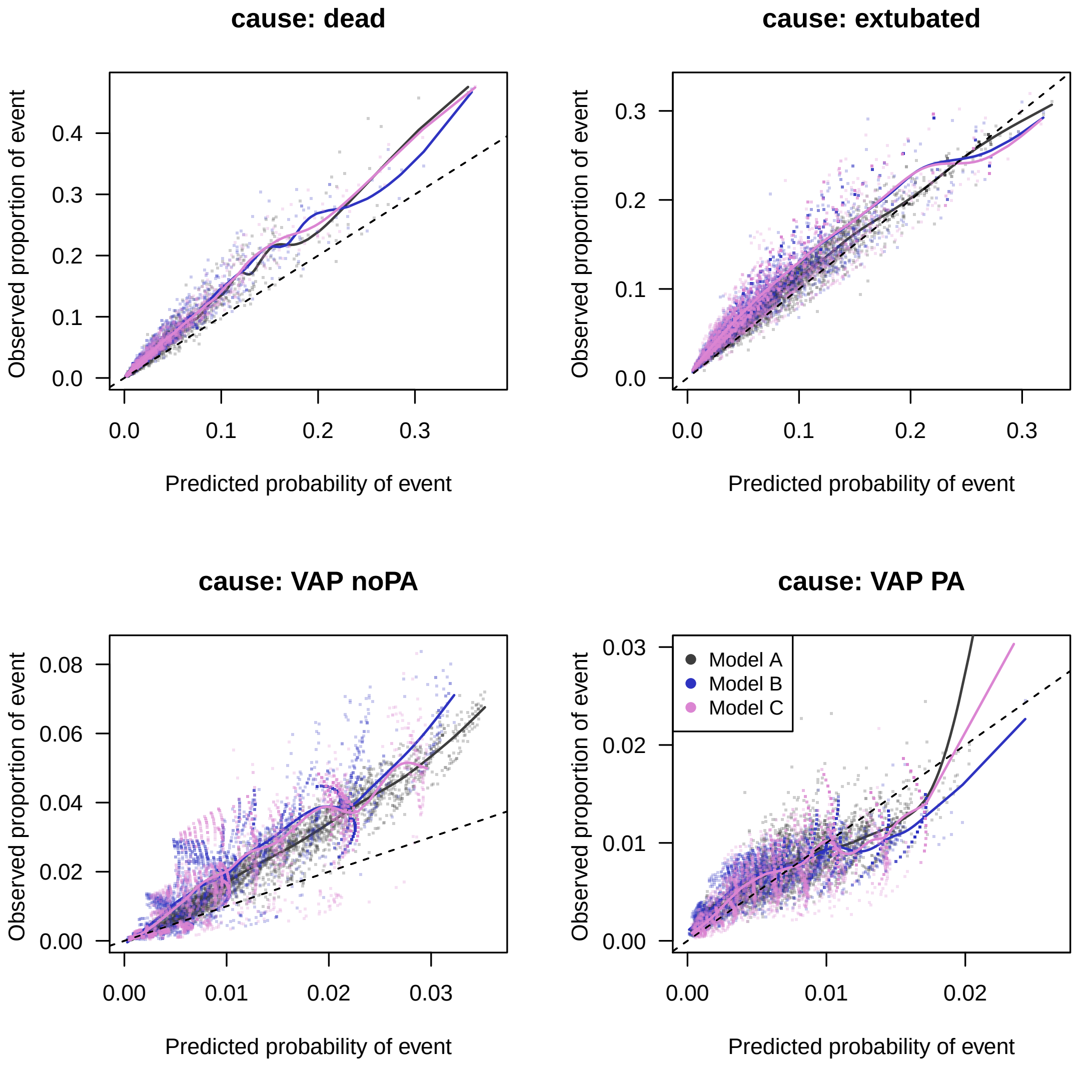

Figure 3 Cause-specific calibration plots for the different models for the predictions of up to 21 days since start of ventilation. Cubic spline smoothers are added for each cause.

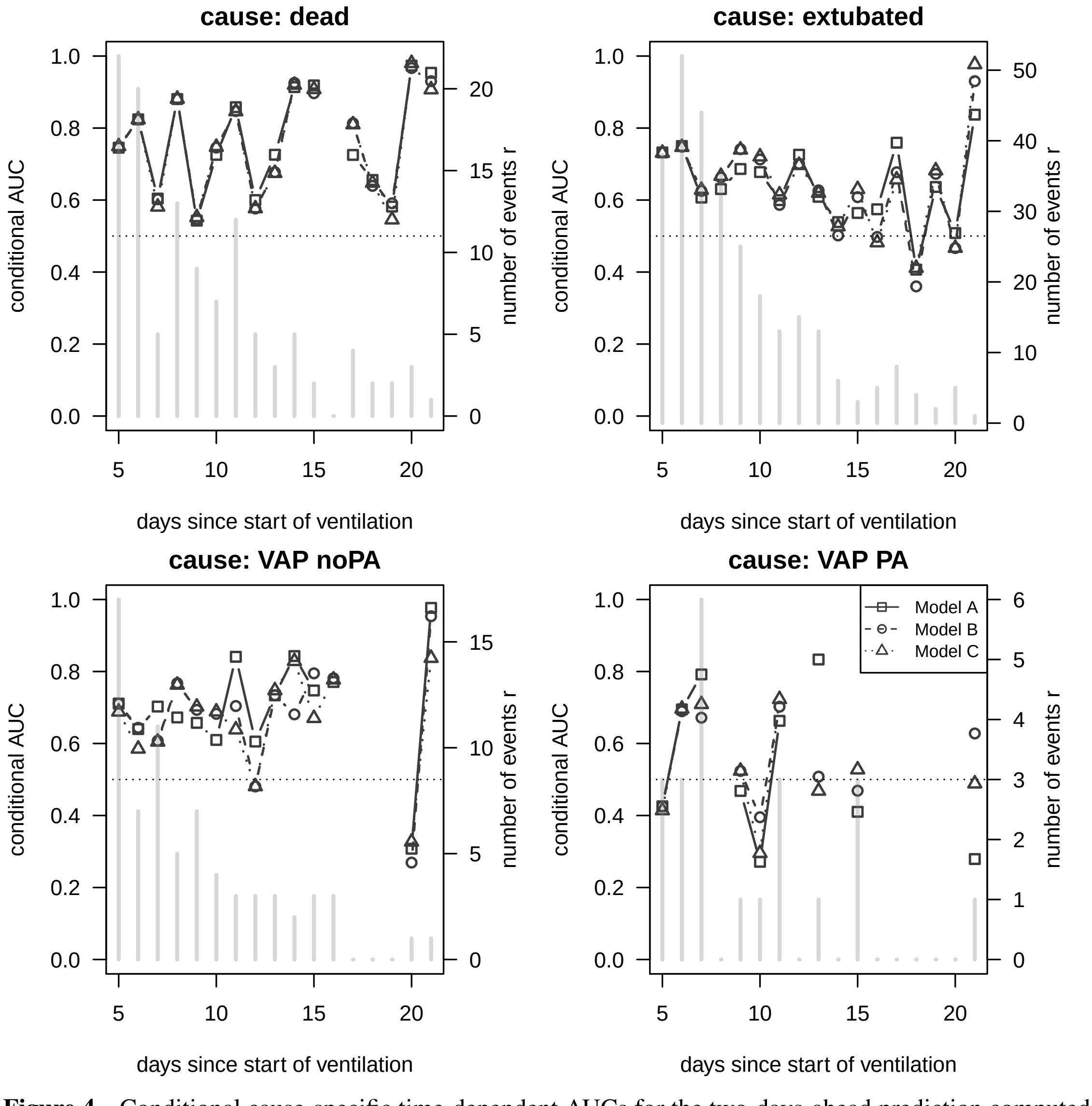

Figure 4 Conditional cause-specific time-dependent AUCs for the two-days-ahead prediction computed with the three models of interest.

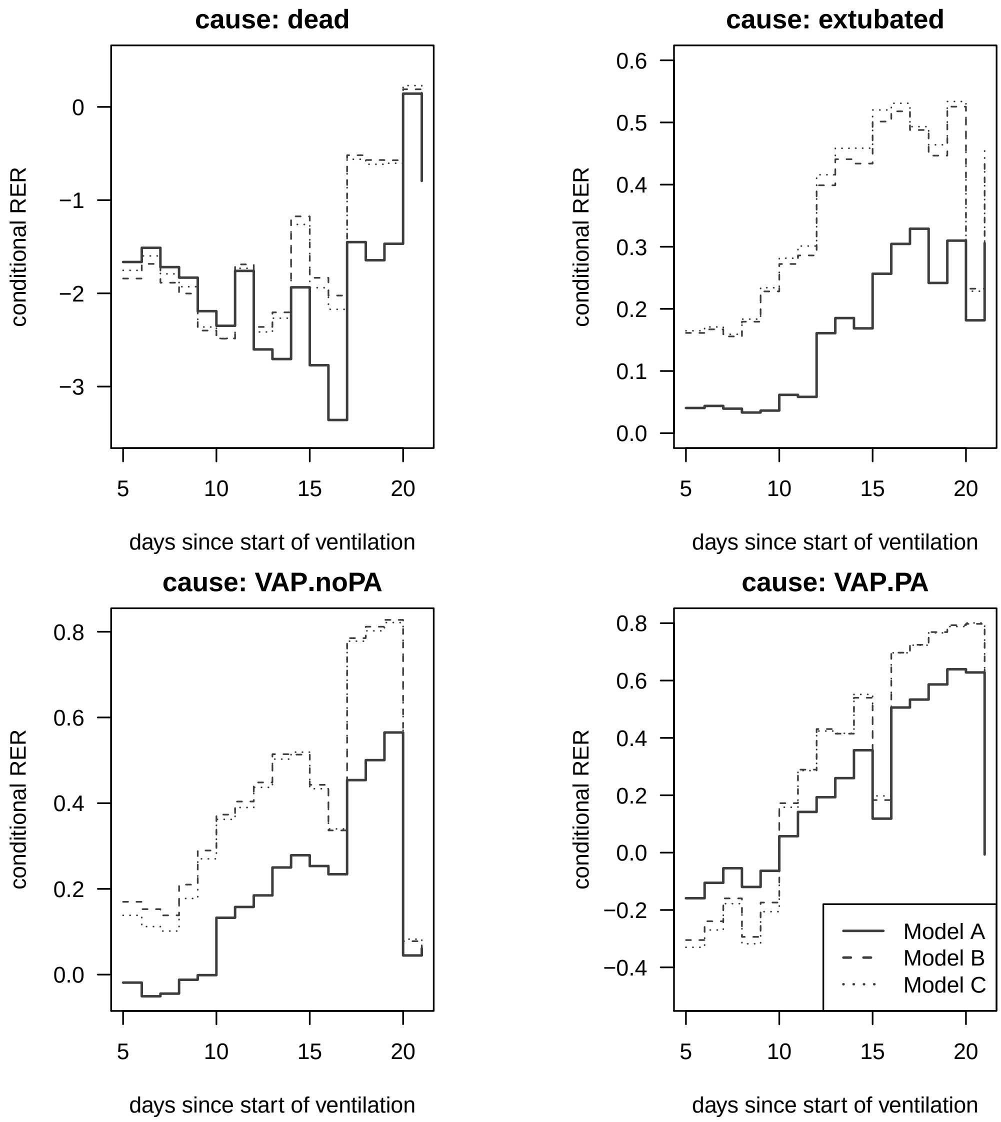

Figure 5 Conditional cause-specific relative error reduction curves for the three candidate models relative to the null model without any covariates.
